# Activities and Role of certified Nurse in Infection Control in COVID-19 Cluster Response in Japan

**DOI:** 10.1017/ash.2024.201

**Published:** 2024-09-16

**Authors:** Masaki Tanabe, Akie Arai, Tomoyuki Uno, Yasuyuki Hara, Kanako Imai, Masumi Tani, Tomoyo Hayashi

**Affiliations:** Mie University Hospital; Mie University Hosptal Department of Medical Health; Mie Prefectural Government; Department of Medical Health, Mie Prefectural Government; Mie Nursing Association, Mie, Japan

## Abstract

**Background:** Mie Prefecture in Japan established a Cluster Response Team within the Headquarters for COVID-19 and registered prefectural staff as well as certified Nurse in Infection Control (CNICs) and other experts, who were promptly dispatched to the site of the cluster and provided other support. However, the extent to which they were dispatched, what activities they performed, and what contributions they made have not been analyzed. **Method:** The Mie prefectural government officials who were responsible for coordinating the dispatch were interviewees regarding the cluster response situation from November 2020 to August 2022. In addition, a questionnaire survey was conducted with CNICs on the supporting side and facility managers on the receiving side regarding the activities and roles of CNICs. **Result:** Of the 275 cluster cases, cluster response teams were dispatched in 59 cases (64% to nursing facilities, 34% to medical institutions). Nineteen of the 46 CNICs registered in Mie Prefecture were dispatched. The number of days CNICs were dispatched ranged from 1 to 4 days, with 1 day being the most common (69.5%). The dispatch coordinators commented that the CNICs they requested were biased, but that they would have liked to request all CNICs to be dispatched. In a survey of CNICs, 36 of 46 (78.3%) responded to the survey. Support was provided for zoning (92%), PPE donning and doffing instruction (92%), infection control evaluation and instruction (85%), cleaning and disinfection services (54%), and training sessions (54%). The tasks that CNIC believed should be performed were generally consistent with the tasks that were actually performed. However, cleaning and disinfection tasks and nursing tasks that were not indicated as tasks to be performed were actually performed. In a questionnaire targeting recipients, 31 of 67 facilities (46.3%) responded to the survey. Respondents indicated that the dispatch of staff improved their knowledge of infection control measures (90.3%), reduced anxiety (87.1%), ensured thorough hand disinfection (61.3%), and standardized the PPE donning and doffing method (58.1%). Requests to the CNIC included regular on-site guidance, sharing and disseminating information, and holding training sessions. **Conclusion:** Administrative staff and infection control staff, mainly CNICs, paired up to provide effective cluster response. However, the uneven distribution of the dispatched CNICs and the unexpected tasks they had to perform indicated the need to re-establish a community-wide infection control system in preparation for the next pandemic.

